# Soluble guanylate cyclase mediates the relaxation of healthy and inflamed bladder smooth muscle by aqueous nitric oxide

**DOI:** 10.3389/fphys.2023.1249560

**Published:** 2023-09-04

**Authors:** Patrik Aronsson, Johanna Stenqvist, Ena Ferizovic, Emelie Danielsson, Anna Jensen, Ulf Simonsen, Michael Winder

**Affiliations:** ^1^ Department of Pharmacology, Institute of Neuroscience and Physiology, The Sahlgrenska Academy, University of Gothenburg, Gothenburg, Sweden; ^2^ Department of Biomedicine, Faculty of Health, University of Aarhus, Aarhus, Denmark

**Keywords:** nitric oxide, bladder, cystitis, relaxation, sGC (soluble guanylate cyclase), NO donor, sodium nitroprusside (SNP), animal study

## Abstract

**Introduction:** Due to its chemical properties, functional responses to nitric oxide (NO) are often difficult to examine. In the present study, we established a method to produce NO in an aqueous solution and validated its capacity to evoke functional responses in isolated rat bladders. Furthermore, we compared the NO responses to the commonly used NO donor sodium nitroprusside (SNP). We also investigated the impact of ongoing inflammation on the involvement of soluble guanylate cyclase (sGC) dependent signaling in NO relaxation.

**Methods:** A setup to produce an aqueous NO solution was established, allowing the production of an aqueous solution containing a calculated NO concentration of 2 mM. Sixty male Sprague-Dawley rats received either no treatment (controls) or cyclophosphamide (CYP; 100 mg*kg^−1^ i.p., 60 h prior to the experiment) to induce experimental cystitis. Bladder strip preparations were mounted in organ baths and studied at basal tension or pre-contracted with methacholine (3 μM). Aqueous NO solution (40–400 μL; 2 mM corresponding to 4–40 μM) or SNP (1–1,000 μM) was added cumulatively in increasing concentrations. Relaxation to aqueous NO was also studied in the presence of the sGC inhibitor ODQ (0.25–25 μM). The expression of sGC was investigated by immunohistochemical analysis.

**Results:** The NO solution caused functional relaxations in both controls and inflamed bladder preparations. NO-induced relaxations were significantly greater in inflamed bladder strips at basal tension, whereas no differences were seen in methacholine pre-contracted strips. In the presence of the sGC inhibitor ODQ in a high concentration, the NO-evoked relaxations were abolished in both control and inflamed preparations. At a lower concentration of ODQ, only NO relaxations in inflamed preparations were attenuated. Immunohistochemical analysis showed that sGC was expressed in the detrusor and mucosa, with a significantly lower expression in the inflamed detrusor.

**Conclusion:** In the present study, we found that aqueous NO solution induces relaxation of the rat detrusor by activating soluble guanylate cyclase in both control and inflamed bladder strips. Induction of inflammation conceivably leads to decreased sGC expression in the detrusor, which may explain the different susceptibility towards inhibition of sGC in inflamed versus control tissue. The use of an aqueous NO solution should be further considered as a valuable complement to the pharmacological tools currently used.

## Introduction

Over the past quarter century, the functional role of nitric oxide (NO) in the lower urinary tract has been outlined. Early on, NO was established as one of the non-adrenergic, non-cholinergic (NANC) signaling molecules released by the urothelium (Winder et al., 2014). A number of studies imply that NO exerts a relaxatory functional effect on the detrusor and modulates afferent signaling (Ozawa et al., 1999; Fujiwara et al., 2000; Andersson et al., 2008; Caremel et al., 2010). However, some studies have indicated that in certain conditions, NO can cause detrusor contractions (Liu and Lin-Shiau, 1997; Moon, 2002; Gillespie and Drake, 2004; Yanai et al., 2008). Apart from via activation of TRPV1 receptors, NO production can also be evoked by stimulation of autonomic receptors. Studies have shown that activation of β-adrenoceptors leads to NO release, mainly from the urothelium (Birder et al., 1998; Winder et al., 2017). During cystitis, urothelial NO production can also be evoked by activation of muscarinic receptors (Andersson et al., 2008; Andersson et al., 2012). The notion of altered nitrergic signaling in inflammation can today be considered an established feature that has been extensively demonstrated in a number of studies (Ozawa et al., 1999; Kang et al., 2004; Andersson et al., 2011; Sancho et al., 2014; Patel et al., 2020).

**FIGURE 1:**
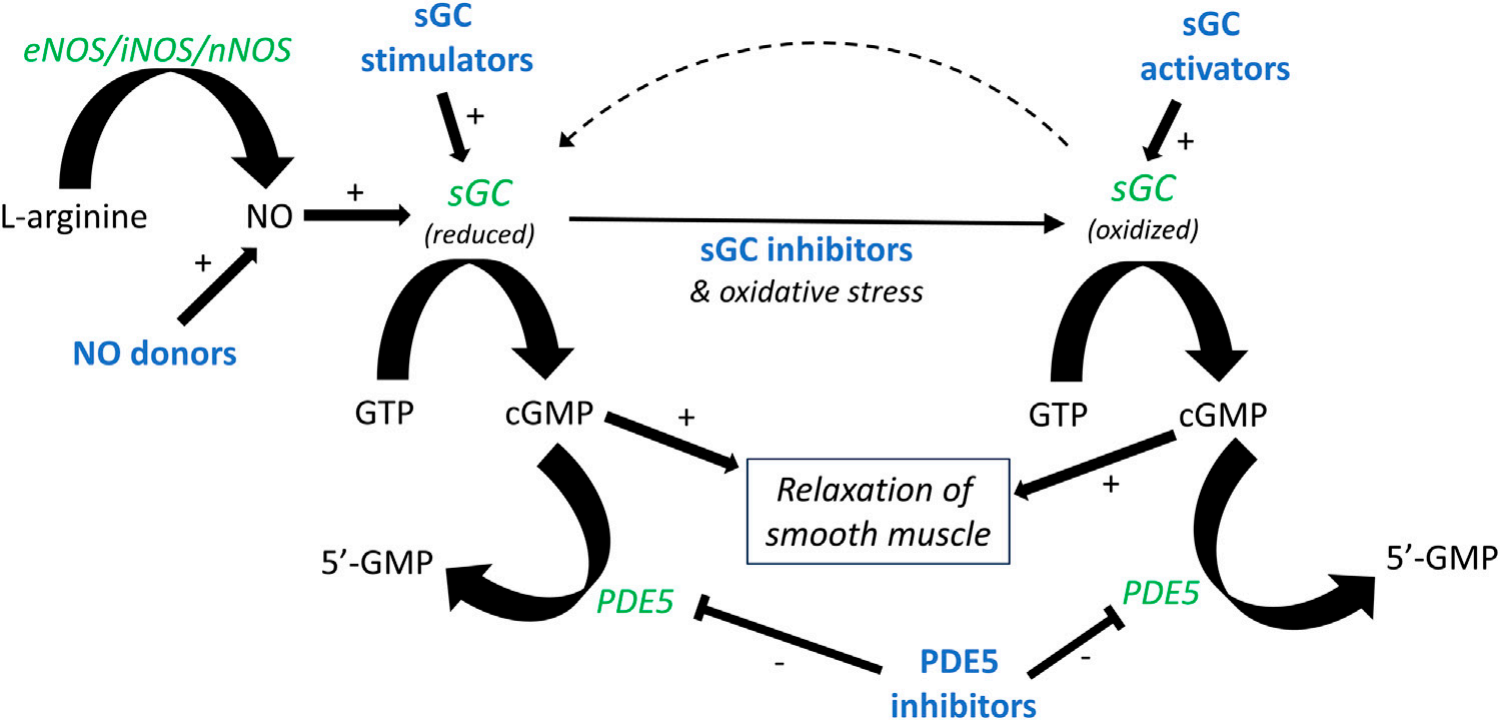
Outline of the NO-sGC-cGMP pathway. The formation of nitric oxide (NO) from L-arginine and molecular oxygen is catalyzed by nitric oxide synthase (NOS). NO binds to soluble guanylate cyclase (sGC), forming cyclic GMP (cGMP) and, in turn, leading to smooth muscle relaxation. Production of cGMP can similarly be induced by NO donors and sGC stimulators. Oxidative stress, for instance during inflammation, leads to oxidation of sGC. A similar effect is achieved by sGC inhibitors such as ODQ. Oxidized sGC cannot be activated by NO, but can instead be activated by sGC activators. Pharmacologically active drugs are illustrated in bold blue, and enzymes in green italics.

Despite the considerable number of studies that have been conducted to outline the functional roles of NO in the lower urinary tract, there are still missing pieces to the puzzle, for instance, an accurate description of which intracellular pathways that are involved in nitrergic signaling. Nevertheless, the involvement of the NO-sGC-cGMP pathway (Figure 1) has repeatedly been demonstrated in basic and clinical studies on various lower urinary tract pathologies (Logadottir et al., 2004; Kedia et al., 2008; Cho et al., 2017; Monica and Antunes, 2018; Zabbarova et al., 2021; Aydogdu et al., 2022). The effects of NO on detrusor contractility have been extensively examined using a wide range of experimental designs. These include *in vivo* (often cystometry), *ex vivo* (organ bath), and *in vitro* (cell cultures) methods (Andersson et al., 2008; Kajioka et al., 2008; Bustamante et al., 2010). Further, studies have utilized different approaches to investigate functional effects of NO. Commonly, either NO donors, such as sodium nitroprusside, nitric oxide synthase inhibitors like L-NNA and L-NAME, or PDE5-inhibitors, such as sildenafil, have been used (Gillespie and Drake, 2004; Andersson et al., 2012; Chakrabarty et al., 2019). Previous studies have in common the non-usage of NO *per se* as an agonist. The main reason for this is the rapid turnover of NO, with an approximate half-life of a few seconds in a normal oxygenated aqueous solution (Moncada et al., 1991). However, in the late 1990s, a method to produce NO in an oxygen-free aqueous solution was established by Simonsen and colleagues at Aarhus University (Feelisch and Kelm, 1991; Simonsen et al., 1999). In brief, pure argon followed by nitric oxide gas is led through pyrogallol and/or sodium hydroxide, removing any traces of oxygen and higher nitrogen oxides, before reaching vials filled with Milli-Q water. Subsequently, NO is dissolved in an aqueous solution capped by inert argon gas. This yields a deoxygenized solution of pure NO in water, thus eliminating the risk of oxidation and breakdown of the substance, which opens the possibility for studies using NO as a classical agonist.

The current study had three interdependent aims. First, to validate the production and use of NO in aqueous solution for studies on bladder functional properties. Second, to study if the relaxations to NO were altered in a state of inflammation. Third, to test the hypothesis that NO in aqueous solution induces bladder relaxation via activation of soluble guanylate cyclase. For these purposes, rats were pre-treated with either saline, serving as control, or cyclophosphamide (CYP; 100 mg*kg^−1^ i.p.), to induce experimental cystitis. NO in aqueous solution was produced according to a previously published protocol (Simonsen et al., 1999). Bladder functional responses to NO in an aqueous solution were examined in a tissue bath setup and compared to those induced by the commonly used NO donor sodium nitroprusside (SNP) in the presence and absence of the soluble guanylate cyclase inhibitor 1H-[1,2,4]oxadiazolo[4,3-a]quinoxalin-1-one (ODQ). Further, the expression of soluble guanylate cyclase was examined by immunohistochemistry.

## Methods and materials

The current study was approved by the local ethics committee at the University of Gothenburg, Sweden (ethical permit 1794/18). A total of 60 male Sprague-Dawley rats (235–440 g) purchased from Charles River (Calco, Italy) were used. Male rats were chosen to allow for comparison to previous studies and to avoid any influence of oestrous cycle on the data. Careful notice was taken to follow all rules and regulations stipulated by the ethical permit, and the number of observations was chosen to obtain sufficient power while minimizing the number of animals included in the study. All experimental procedures had full adherence to the ARRIVE 2.0 guidelines.

### Experimental design and tissue preparation

The rats were housed under standard conditions with access to food and water *ad libitum*. Animals belonging to the control group received no treatment, whereas experimental cystitis was induced through a single injection of cyclophosphamide (CYP, 100 mg/kg i.p.) 60 h prior to the experimental procedures, ensuring peak inflammation at the time of sacrifice (Giglio et al., 2005), when anaesthesia and euthanasia were induced by an overdose of pentobarbitone (>60 mg/kg i.p.; APL, Stockholm, Sweden). The pelvic cavity was opened, and the urinary bladder was excised and placed in Krebs solution (composition: see section “Tissue bath experiments”). To ensure euthanization, a subsequent excision of the heart was performed.

Full-thickness bladder strips (2 mm × 6 mm) were cut from above the trigone and proximal to the ureters according to a standard procedure (Aronsson et al., 2010) and mounted in organ baths (see below). 1-2 strips were taken from each rat bladder.

### Production of aqueous nitric oxide

**FIGURE 2:**
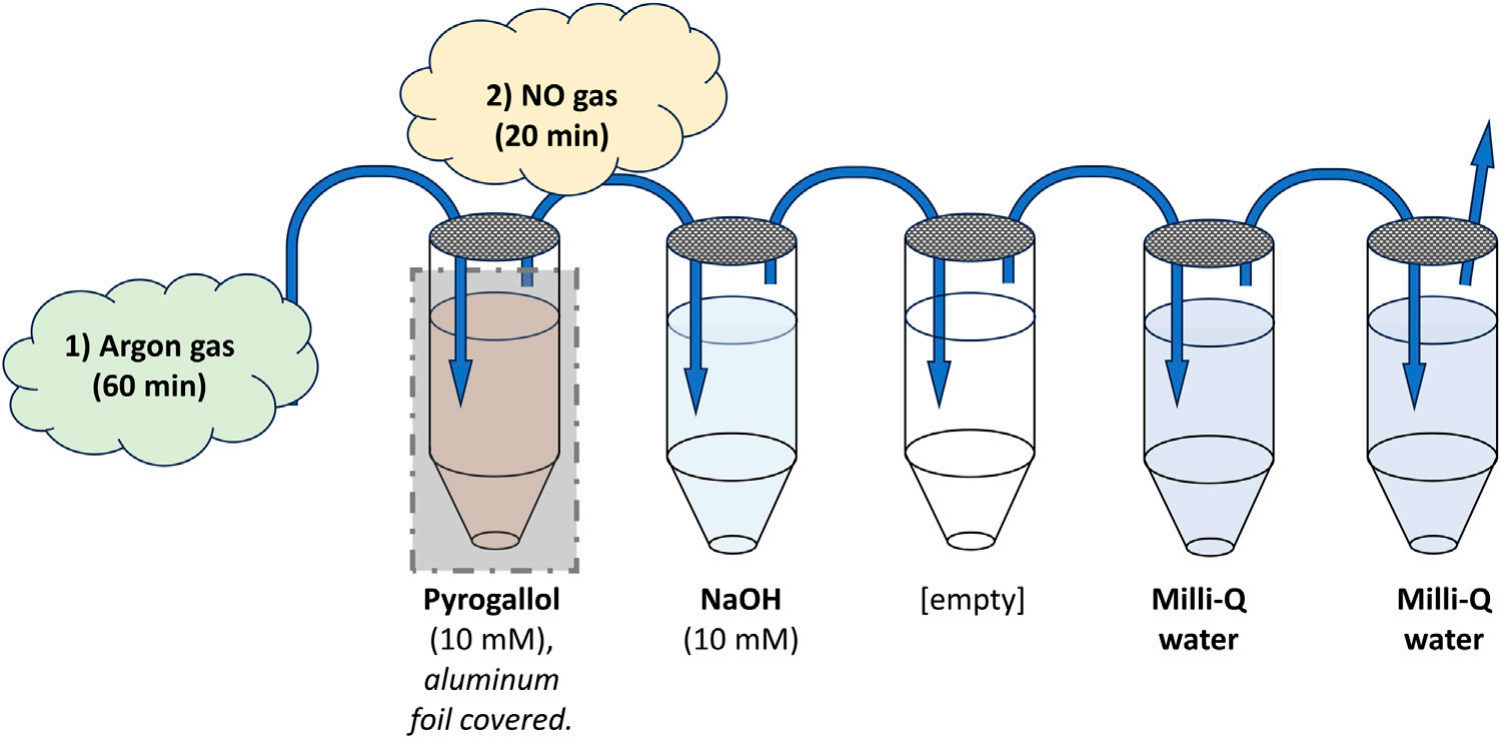
Schematic illustration of the setup for producing the aqueous NO solution. Five 20 mL glass vials with septum closure were set up in a fume hood. The first was filled with 18.5 mL of pyrogallol (10 mM) to remove any traces of oxygen from the argon gas led through it. The next contained 18.5 mL of NaOH (10 mM) to remove higher nitrogen species from any gas flowing through it. Thereafter, an empty vial was placed to ensure no spillover into the final vials containing pure Milli-Q water. All vials were connected with Teflon tubing (blue arrows), entering each vial into the liquid and exiting above. The final vial had a Teflon tube leading into the open air, functioning as a “chimney” for the supplied gas. To minimize oxidization, all solutions were prepared directly in the glass vials, and the pyrogallol vial was covered with aluminum foil due to its photosensitive nature. Argon gas, pushing out oxygen from the solutions and vials, was first led through the system for 60 min. Then the gas was switched, without interruption, to pure NO gas, entering through the NaOH vial, for an additional 20 min. The end result was two vials of Milli-Q water saturated with pure NO.

An aqueous NO solution with a calculated concentration of 2 mM, based on NO solubility, and a shelf-life of up to 1 week was prepared (Simonsen et al., 1999). As schematically shown in Figure 2, a system with five 20 mL glass vials with septum closure was set up in a fume hood; the first containing pyrogallol (10 mM), the next NaOH (10 mM), then an empty vial, and finally two vials filled with Milli-Q water. To minimize oxidization, all solutions were prepared directly in the glass vials, and the pyrogallol vial was covered with aluminum foil due to its photosensitive nature. The vials were connected in sequence with Teflon tubes with flowing argon gas, pushing out oxygen from the solutions. After all vials had been connected, the argon flow was kept for 1 h and then switched to pure NO gas, entering the NaOH vial, without interruption for an additional 20 min. The final vial had a Teflon tube leading into the open air, functioning as a “chimney” for the supplied gas. The same procedure was conducted in a pilot experiment, except for the final gassing with NO, serving as negative control (vehicle) for validation purposes.

### Tissue bath experiments

The full-thickness bladder strip preparations were mounted between two steel rods, one adjustable and one fixed, connected to an isometric force transducer (TSD125C, Biopac Systems Inc., Goleta, United States) and immersed into organ baths (20 mL) filled with Krebs solution [NaCl, 118 mM; KCl, 4.6 mM; KH_2_PO_4_, 1.15 mM; MgSO_4_ (anhydrous), 1.15 mM; NaHCO_3_, 25 mM; CaCl_2_, 1.25 mM; and glucose, 5.5 mM] gassed with 95% O_2_ and 5% CO_2_ at 37°C. The bladder strip preparations were stretched to 10 mN and let to equilibrate, resulting in a stable tension of about 5–7 mN after 45 min. The resulting tension was the starting point for measurements of responses to subsequent drug administration at basal tension.

The viability of the preparations was assessed by an administration of methacholine (3 × 10^−5^ M), after which the tissue was washed twice and left to equilibrate for 20 min. Pre-contraction of the tissue was achieved, where applicable, by administration of methacholine (3 × 10^−6^ M). In a few pilot experiments, high potassium Krebs solution (50 mM; sodium exchanged for potassium) was instead used to achieve pre-contraction. However, the results were identical to those when using methacholine (data not shown).

All administrations were given at a volume of 100 µL using a micropipette, except for the NO solution (or corresponding vehicle in a few control experiments), which was administered with a gas-tight Hamilton syringe in close proximity to the tissue. The NO solution was produced in a set concentration (2 mM) and administered in volumes of 40, 100, 200, and 400 μL, resulting in the desired final concentrations (4 × 10^−6^, 10^-5^, 2 × 10^−5^, and 4 × 10^−5^ M).

The soluble guanylyl cyclase inhibitor 1H-[1,2,4]oxadiazolo[4,3-a]quinoxalin-1-one (ODQ; 2.5 × 10^−7^–2.5 × 10^−5^ M) was, where applicable, let to equilibrate for 20 min before the addition of any agonist, i.e., NO solution or NO donor (SNP). Before adding NO or NO donor, the tissues were pre-contracted with methacholine (3 × 10^−6^ M). The concentrations of ODQ were chosen based on previous studies in the literature and observations from pilot studies (Artim et al., 2009).

All concentrations stated are the resulting concentrations in the organ baths, and subsequent administrations were done cumulatively. All substances were diluted in Milli-Q water.

The following substances were used: pentobarbitone (APL, Stockholm, Sweden), argon gas (Linde Gas AB, Enköping, Sweden), NO gas (Linde Gas AB, Enköping, Sweden), pyrogallol, sodium hydroxide (NaOH), sodium chloride (NaCl), potassium chloride (KCl), potassium dihydrogen phosphate (KH_2_PO_4_), magnesium sulphate (MgSO_4_), sodium bicarbonate (NaHCO_3_), calcium chloride (CaCl_2_), glucose (C_6_H_12_O_6_), cyclophosphamide, sodium-nitroprusside (SNP), 1H-[1,2,4]oxadiazolo[4,3-a]quinoxalin-1-one (ODQ; Bio-techne, Minneapolis, United States) and methacholine. All substances were purchased from Sigma-Aldrich (St Louis, United States) unless otherwise stated.

### Immunofluorescence

Expression of soluble guanylate cyclase (sGC) was examined by fluorescent immunohistochemistry. Immediately following an organ bath experiment, the tissues were fixed in paraformaldehyde (4% in 0.1 M phosphate buffered saline). Thereafter, the tissues were embedded in paraffin and sectioned into 10 µm thin tissue sections (two sections per glass; Histolab, Gothenburg, Sweden). The immunofluorescent staining procedure was then commenced by deparaffinizing the paraffin-embedded tissues in xylene, followed by rehydration in 99.5%, 95%, 70%, and 50% ethanol. To remove autofluorescence, copper(II) sulfate (CuSO_4_, 1 mM; pH = 5) was added to each section for 10 min. Thereafter, heat-induced epitope retrieval was achieved by heating the sections to 70°C in sodium citrate buffer (pH 5.0; Sigma-Aldrich) in a microwave oven for 30 min. Upon reaching room temperature, a blocking solution (5% goat serum, Vector Laboratories, Burlingame, United States and 0.25% Triton X-100, Thermo Fisher Scientific, Rockford, IL, United States, in PBS) was added for 1 h. Next, the primary rabbit polyclonal anti-sGC beta 2 antibody was added to one section on each glass (50 μL; ab53084, Abcam, Cambridge, United Kingdom; 1:100 in 1% goat serum and 0.25% Triton X-100, in PBS), while the other section was kept as a negative control (50 μL; 1% goat serum and 0.25% Triton X-100, in PBS; no antibody). The sections were thereafter incubated overnight at 4°C. The following day, a secondary antibody (ab6719, Abcam, Cambridge, United Kingdom; 1:500 in 1% goat serum and 0.25% Triton, in PBS) was added to the sections and let to incubate for 1 h at room temperature. The sections were thereafter dehydrated in 50%, 70%, 90%, and 99.5% ethanol, respectively. Finally, the sections were mounted under a cover glass with ProLong Gold antifade reagent with DAPI (Thermo Fischer Scientific, Eugene, United States).

### Data analysis and statistics

Statistical calculations were performed using GraphPad Prism version 9.5.0 (GraphPad Software Inc., San Diego, United States). Two-way ANOVA followed by Šidák`s correction for multiple comparisons was used for statistical comparisons of tissue bath data, i.e., of concentration-response curves. Statistical significance was regarded for *p*-values < 0.05. Data are presented as mean ± SEM.

Protein expression was analyzed semi-quantitatively by grading the expression of sGC in each tissue on a scale from 0 to 3 where 0 = no protein expression, 1 = weak protein expression, 2 = moderate protein expression and 3 = strong protein expression. The grading was performed by three blinded evaluators (MW, ED, JS) on images taken with a DS-Fi camera mounted in a Nikon 90i fluorescence microscope (Nikon Corporation, Tokyo, Japan). All images were taken after digital subtraction of background staining measured in corresponding negative controls (i.e., in which the primary antbody was excluded), using the NIS Element Imaging Software v. 4.40 (Nikon Corporation). Each evaluator graded each tissue. Subsequently, a consensus grade was determined for each tissue. Thereafter, the tissues were decoded and a non-parametric statistical calculation was carried out by running a Mann-Whitney rank test.

## Results

### Relaxation of bladder basal tension

**FIGURE 3:**
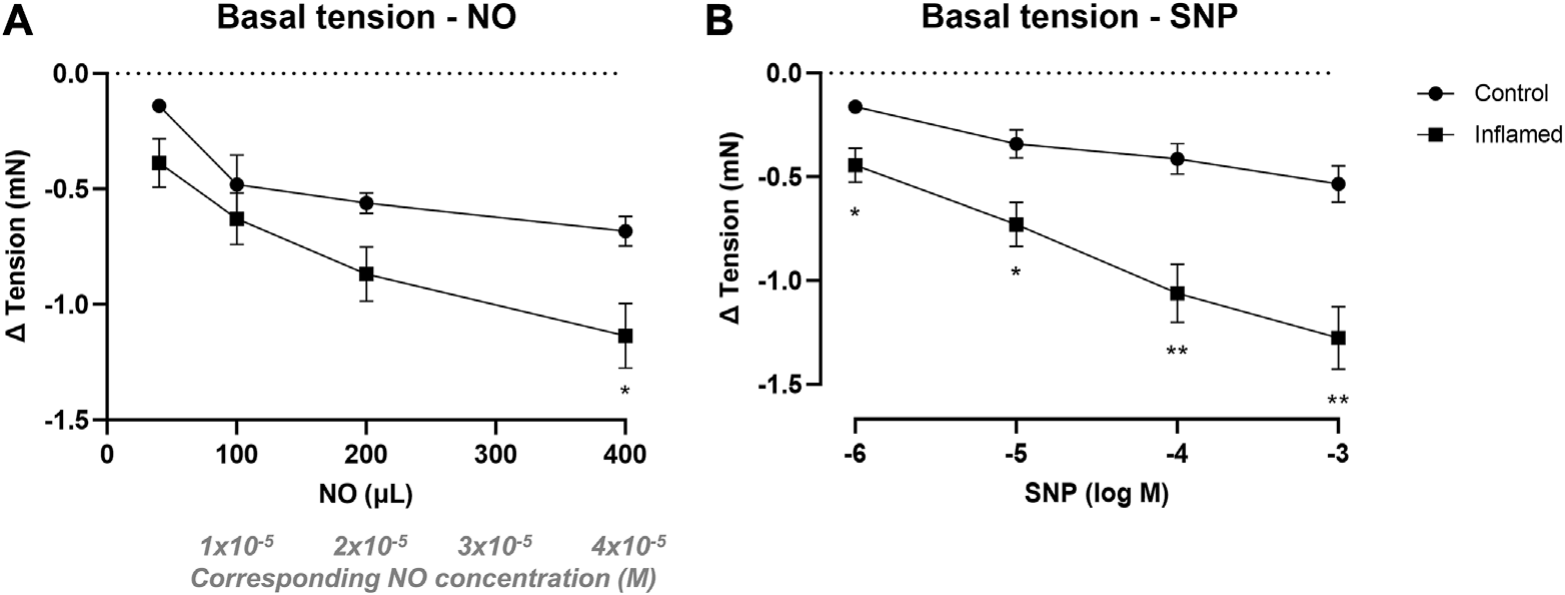
Nitrergic relaxation of basal tension in bladder preparations. Functional relaxatory responses at mechanically attained basal tension to increasing volumes of **(A)** aqueous NO solution or concentrations of **(B)** the NO donor SNP in full-thickness urinary bladder strip preparations from healthy controls (•) or rats with cyclophosphamide-induced cystitis (▪). * and ** denotes *p* < 0.05 and *p* < 0.01, respectively. *n* = 8 in each group. Vertical bars indicate the SEM.

NO in aqueous solution (40–400 μL; 2 mM corresponding to 4 × 10^−6^, 1 × 10^−5^, 2 × 10^−5^, and 4 × 10^−5^ M) induced concentration-dependent relaxation in both healthy and inflamed detrusor strips (Figure 3A). The relaxations were significantly greater in inflamed tissues when adding the largest volume of NO (i.e., at 400 μL; −0.68 ± 0.06 mN and −1.14 ± 0.14 mN in healthy and inflamed detrusor strips, respectively; *p* = 0.039; Figure 3A). Similarly, SNP (10^−6^–10^−3^ M) induced concentration-dependent relaxation of basal tension in both healthy and inflamed detrusor strips (Figure 3B). The relaxations were significantly greater in inflamed tissues at all SNP concentrations (1–1,000 μM; *p* < 0.05; Figure 3B). The addition of vehicle (pilot experiments), i.e., Milli-Q water prepared in the same way as the NO-solution but in the absence of NO, did not produce any functional responses in neither healthy nor inflamed tissues (data not shown).

### Relaxation of pre-contracted bladder tissue

**FIGURE 4:**
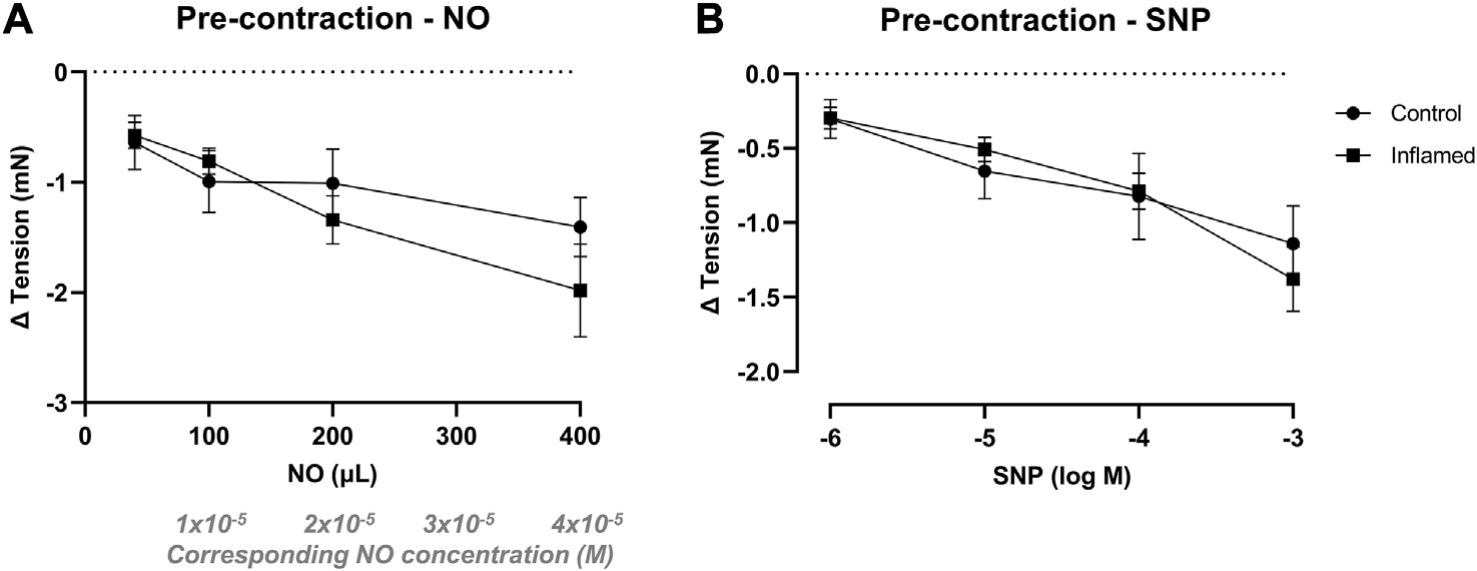
Nitrergic relaxation of pre-contracted bladder preparations. Functional relaxatory responses to increasing volumes of **(A)** aqueous NO solution or concentrations of **(B)** the NO donor SNP in methacholine pre-contracted full-thickness urinary bladder strip preparations from healthy controls (•) or rats with cyclophosphamide-induced cystitis (▪). *n* = 8 in each group. Vertical bars indicate the SEM.

In preparations contracted with methacholine (3 × 10^−6^ M), NO in aqueous solution (40–400 μL; 2 mM) induced concentration-dependent relaxations in detrusor strips from both healthy and inflamed bladders (from −0.64 ± 0.25 to −1.41 ± 0.27 and from −0.57 ± 0.12 to −1.98 ± 0.42 mN in healthy and inflamed tissues, respectively; Figure 4A). SNP (10^−6^–10^−3^ M) induced similar concentration-dependent relaxations in pre-contracted detrusor strips from both healthy and inflamed bladders (from −0.30 ± 0.13 to −1.14 ± 0.25 and from −0.30 ± 0.07 to −1.38 ± 0.22 mN in healthy and inflamed tissues, respectively; Figure 4B). There were no significant differences in the relaxations to neither NO nor SNP in healthy vs. inflamed detrusor strips (Figures 4A, B).

### Inhibition of bladder relaxation via soluble guanylate cyclase

**FIGURE 5:**
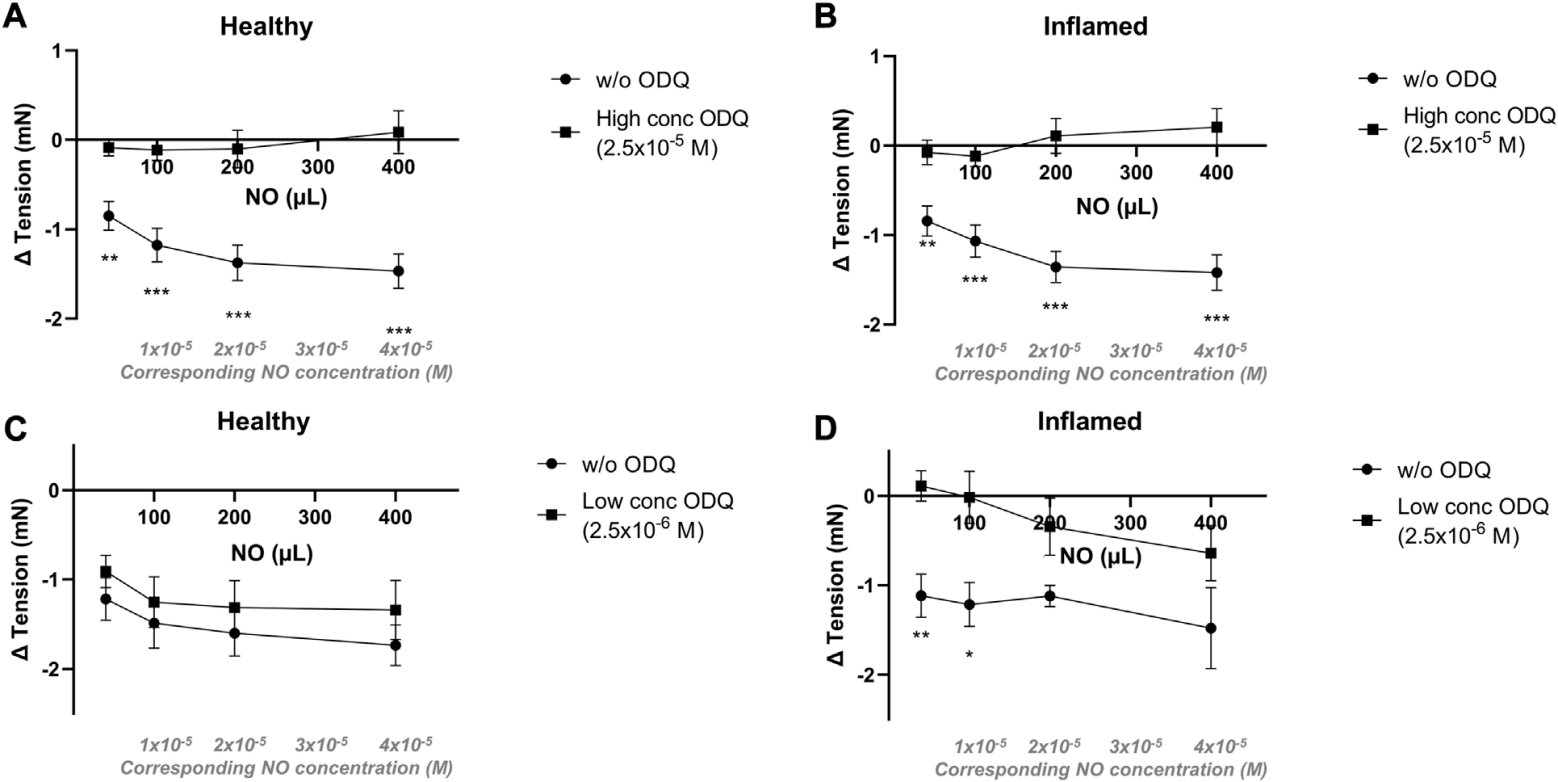
Effects of the sGC inhibitor ODQ on nitrergic relaxatory responses in pre-contracted bladder preparations. Nitrergic relaxations to increasing volumes of aqueous NO solution in the absence (•) or presence (▪) of ODQ in full-thickness urinary bladder strip preparations from healthy controls **(A,C)** or rats with cyclophosphamide-induced cystitis **(B,D)**. In the upper panels **(A,B)**, the ODQ concentrations used were high (2.5 × 10^−5^ M), and in the lower panels **(C,D)**, the concentrations were low (2.5 × 10^−6^ M) in the lower. *, **, and *** denotes *p* < 0.05, *p* < 0.01, and *p* < 0.001, respectively. *n* = 11 in each group. Vertical bars indicate the SEM.

Relaxations to NO in aqueous solution were completely abolished in both healthy and inflamed tissues in the presence of a high concentration (2.5 × 10^−5^ M) of the soluble guanylate cyclase inhibitor ODQ (*p* < 0.01 at all concentrations; Figures 5A, B). In the presence of a low concentration (2.5 × 10^−6^ M) of ODQ, the relaxations to NO remained intact in healthy tissues (*p* > 0.05 at all concentrations; Figure 5C). However, in inflamed bladder strip preparations, a low concentration of ODQ significantly attenuated the relaxations to lower but not higher concentrations of NO (*p* = 0.0023 and 0.021 at 40 and 100 µL of a 2 mM aqueous solution, corresponding to NO concentrations of 4 × 10^−6^ and 1 × 10^−5^ M, respectively; Figure 5D). No blocking of the relaxations to NO was observed in the presence of an additionally lower concentration of ODQ (i.e., 2.5 × 10^−7^ M; data not shown).

### Expression of soluble guanylate cyclase

**FIGURE 6:**
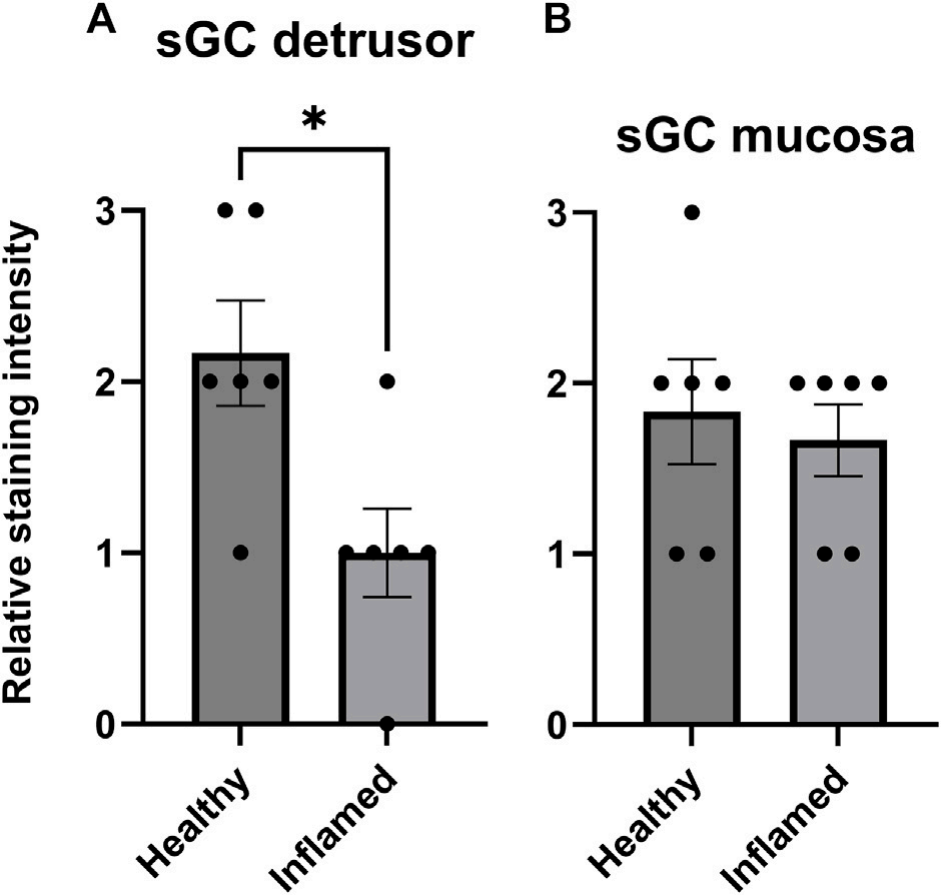
Expression of sGC in the rat detrusor and urothelium. Comparison of fluorescent sGC expression in healthy and inflamed **(A)** detrusor and **(B)** urothelium. Expression was graded on a relative scale from 0–3 where 0 denoted no expression and 3 denoted maximum fluorescent intensity. *n* = 6 in each group; each individual grade is indicated by a black point in the figure. *p < 0.05.

The immunohistochemical analysis showed soluble guanylate cyclase (sGC) expression in both the detrusor and mucosa in all tissues (Figure 6). The semi-quantitative analysis revealed a significantly lower expression of sGC in the inflamed detrusor than in healthy tissues (*p* = 0.023; Figure 6A). No significant differences in sGC expression were observed in the mucosa (Figure 6B).

## Discussion

The current study demonstrates the usefulness of NO in aqueous solution when studying contractile smooth muscle responses in a tissue bath setup. No differences between functional responses to NO in aqueous solution and those of SNP could be seen. Thus, the NO solution and SNP can be used interchangeably. However, NO in aqueous solution has advantageous properties compared to SNP. Most importantly, the amount of freely available NO is known and can exert its effect immediately upon addition to the tissue bath. When adding SNP, the amount of freely available NO is unknown and dependent on the cleavage rate of the parent drug. This pharmacokinetic component is avoided when using NO in an aqueous solution, thus mimicking the physiological situation more closely. This does not limit the use of SNP as a pharmacological substance. Ideally, both aqueous NO and NO donors can be used in parallel in experimental setups. It would also be essential to examine NO in aqueous solution for use *in vivo*, e.g., for instillation in the bladder, which should be addressed in future studies.

When measuring nitrergic responses at basal tension, the relaxations to aqueous NO and, in particular, SNP were greater in inflamed tissues as compared to healthy tissues. This indicates that the inflamed tissue is more sensitive to nitrergic relaxation when in a resting, uncontracted state than normal tissue. When considering that induction of inflammation reduces the expression of sGC in the detrusor, as well as potentially increases oxidation of sGC, this is a surprising finding. In contrast, the relaxations to both NO in aqueous solution and SNP were similar in methacholine pre-contracted healthy and inflamed tissues. This finding is also quite surprising, considering that several previous studies have shown that significant changes regarding nitrergic signaling arise upon induction of inflammation (Andersson et al., 2008; de Oliveira et al., 2016). Apparently, muscarinic receptor-mediated contraction still allows NO to exert its relaxatory effects, but manages to mask the differences between bladder preparations from normal rats and rats with cystitis. The mechanism behind this is presently left unexplained but may emanate from the higher tensions involved or a variety of receptor subtypes being activated by the agonist. This is interesting and should be investigated further, but nonetheless, the current data indicate that responses to NO, and its effects on sGC, remain the same also in the inflamed bladder.

It has long been known that NO induces smooth muscle relaxation by stimulating the formation of cyclic GMP (cGMP) (Kukovetz et al., 1987). It does so by binding to the heme moiety in sGC. However, NO can only bind to heme in its reduced state. ODQ blocks sGC by oxidizing the heme group (Zhao et al., 2000), thus disrupting the ability of NO to bind. In the current study, a higher concentration of ODQ led to the total abolishment of NO-induced relaxations in both healthy and inflamed tissues. In the presence of a lower concentration of ODQ (2.5 × 10^−7^ M), the inhibition of NO-induced relaxation was absent. Thus, the inhibitory effect of ODQ was concentration-dependent and specific, demonstrating that NO-induced detrusor relaxation occurs via the activation of sGC and the subsequent formation of cGMP. This is in line with previous findings in other disease models (Bau et al., 2010; Fullhase et al., 2015). It should be noted that inflammation is a common cause of oxidative stress, which can lead to oxidation of the heme moiety in sGC. However, the current data clearly show that despite bladder inflammation, NO can still induce smooth muscle relaxation via sGC. Thus, the present findings indicate that induction of bladder inflammation with CYP does not induce substantial oxidative stress, at least not above the threshold, allowing sGC to remain functional. However, the amounts of NO used in the current study to induce detrusor relaxation may be greater than what is produced *in vivo*. Considering that several previous studies have demonstrated that impairment of the NO-sGC-cGMP pathway is strongly associated with lower urinary tract symptoms, and that restoring this pathway ameliorates symptoms (Monica and Antunes, 2018; Aydogdu et al., 2022), the current data should be interpreted with this in mind. It should also be noted that levels of cGMP were not measured in the current study.

The immunofluorescent analysis showed that sGC is expressed throughout the bladder wall, albeit with a relatively low level of expression in the submucosa. Mucosal, i.e., mainly urothelial, expression was similar when comparing healthy and inflamed tissues. However, upon induction of inflammation, the expression of sGC was attenuated in the detrusor. This aligns with previous studies in various lower urinary tract disease models showing decreased sGC expression upon induction of bladder dysfunction (Fullhase et al., 2015; de Oliveira et al., 2016). The currently used primary antibody cannot distinguish between reduced and oxidized sGC. However, the immunostainings indicated a lower level of expression of sGC in the inflamed detrusor. When considering the blocking ability of ODQ in healthy as compared to inflamed tissues, the data could be interpreted as ODQ in a lower concentration (2.5 × 10^−6^ M; Figures 5C, D) being able to block NO-induced relaxation in inflamed tissues but not in healthy as a result of a lower amount of sGC, or a fraction of sGC already being oxidized, in the inflamed detrusor. The immunofluorescent analysis thus supports the findings in the tissue baths regarding relaxations to NO in the presence of ODQ.

Even though the present study validated the method of producing and employing NO in aqueous solution, a few experimental challenges are worth noting. First, the administration of the NO solution must be performed in close proximity to the preparation in the organ bath to avoid rapid breakdown. While this could theoretically be an issue, the current results demonstrate concentration-dependent effects, indicating a proper diffusion of the substance. Second, since the aqueous NO solution is produced in a set concentration, the volume administered will increase with the intended concentration. This was found not to present any problem experimentally since administration of the vehicle in the relevant volumes did not alter the tension of the preparations.

Future studies should be designed to examine the intracellular pathways in play upon activation of sGC, including the resulting amounts of cGMP. Various time points during cystitis development should be investigated to further unravel bladder alterations due to inflammation. This would increase the understanding of nitrergic signaling in the urinary bladder. It would also be beneficial to specifically examine activation of sGC in its oxidized form, for example, by utilizing sGC activators. If simultaneously examining responses to NO, it may be possible to quantify the functional proportion of sGC, i.e., the amount of reduced vs. oxidized sGC, in different disease states.

## Conclusion

In the present study, we found that aqueous NO solution induces relaxation of the rat detrusor by activating soluble guanylate cyclase in both control and inflamed bladder strips. Induction of inflammation conceivably leads to decreased sGC expression in the detrusor, which may explain the different susceptibility towards inhibition of sGC in inflamed versus control tissue. Further, the current findings verify the usefulness of utilizing NO in aqueous solution for studies of the lower urinary tract, indicating this to be a good complement to currently used pharmacological tools.

## Data Availability

The raw data supporting the conclusion of this article will be made available by the authors, without undue reservation.
